# Delay in Accessing and Receiving Primary Health Care and Associated Factors Among Nepalese Immigrant Patients in Canada

**DOI:** 10.3390/healthcare14020252

**Published:** 2026-01-20

**Authors:** Bishnu B. Bajgain, Mohammad Z. I. Chowdhury, Rudra Dahal, Kalpana Thapa Bajgain, Kamala Adhikari, Nashit Chowdhury, Tanvir C. Turin

**Affiliations:** 1 Department of Community Health Sciences, Cumming School of Medicine, University of Calgary, Calgary, AB T2N 4N1, Canada; bishnu.bajgain@ucalgary.ca (B.B.B.);; 2 Community Scholar and Citizen Researcher, Nepalese-Canadian Community, Calgary, AB T2N 1N4, Canada; 3 Department of Family Medicine, University of Calgary, Calgary, AB T2N 4N1, Canada; mohammad.chowdhury@ucalgary.ca; 4 Department of Population and Public Health, Alberta Health Services, Calgary, AB T2W 1S7, Canada

**Keywords:** delay in care, primary healthcare, healthcare system, access, barriers, equity, Nepalese, immigrant

## Abstract

**Introduction**: Timely access to healthcare is essential for improving population health and reducing inequities. Immigrants often experience unique cultural, linguistic, and systemic barriers that delay care-seeking and service utilization. Despite the rapid growth of the Nepalese community in Canada, there is limited empirical evidence examining their healthcare access. This study aimed to assess the prevalence and determinants of delays in accessing healthcare among Nepalese immigrants. **Methods**: A community-based participatory research (CBPR) framework guided a cross-sectional survey conducted between January and June 2019. The research process was co-led by academic investigators, community scholars, and local Nepalese organizations to ensure cultural and contextual relevance. A snowball sampling strategy was used to recruit 401 Nepalese adults. Data were analyzed using descriptive statistics and multivariable logistic regression to examine sociodemographic and health-related factors associated with delayed healthcare access. **Results**: Of the 401 respondents, 66.3% (n = 266) reported experiencing a delay in accessing healthcare within the preceding 12 months. Delays were more common among participants aged 26–45 years, those who were married, employed, or had an undergraduate degree or lower. After adjusting for covariates, older age, lower education, having a family doctor, higher income (≥$26,000), and one or more chronic conditions were associated with increased odds of delay. Family size and the number of years living in Canada have had little effect on care delay. **Conclusions**: Delays in accessing healthcare are common among Nepalese immigrants in Calgary, reflecting the intersection of individual, cultural, and systemic determinants. These findings underscore the importance of community-engaged, culturally responsive strategies to address barriers and promote equitable healthcare access for immigrant populations. Strengthening partnerships between health systems and immigrant communities may enhance trust, navigation, and continuity of care.

## 1. Introduction

Primary Health Care (PHC) serves as the main entry point to the healthcare system for most Canadians seeking medical services in Canada. In Canada, each province and territory organizes healthcare services according to its own context through independently managed health insurance plans. Nevertheless, all jurisdictions adhere to the fundamental principles of the Canada Health Act, universality, transferability, comprehensiveness, accessibility, and public administration.

Easy and timely access to healthcare services is a critical determinant of overall health. It represents a dynamic process shaped by individuals’ health-seeking behaviors and the efficiency of healthcare delivery systems. Equitable access to healthcare, regardless of socioeconomic status or place of residence, remains essential [1]. Difficulties in accessing care can lead to delays in seeking or receiving treatment, thereby increasing the risk of morbidity and mortality associated with otherwise treatable or preventable conditions. Delay in accessing care refers to experiencing a wait or difficulty in obtaining timely medical attention from providers after deciding to seek help.

The link between delays in seeking or accessing healthcare and poor health outcomes is well established [2,3]. Delays in care-seeking can reduce access to timely diagnosis and treatment, resulting in suboptimal outcomes [3]. Multiple factors contribute to such delays, including availability, long waiting time, funding of services, attitudes toward healthcare, cultural and language barriers, social support, difficulties navigating the healthcare system, health insurance coverage, employment responsibilities, and preferences for specific providers [3,4,5].

Timely access to healthcare services is vital for the health of all Canadians, including immigrants. When individuals face barriers to accessing care or delay seeking and receiving appropriate services, whether due to underuse or lack of awareness of preventive care, the consequences may include increased risk of complications and a higher economic burden on the healthcare system (e.g., longer hospital stays or more intensive treatment needs) [3,6,7,8]. The goal of Canada’s universal health insurance program is to ensure that all residents have access to medically necessary services free of direct cost [9]. However, immigrants often encounter barriers such as language differences, cultural unfamiliarity, health system navigation challenges, costs, and extended wait times, which contribute to delays in accessing PHC [3,5,6].

Immigrants, a person born outside Canada and has been granted the right to live in Canada permanently, comprise more than one-fifth (21.9%) of Canada’s population, a figure projected to rise to 30% by 2036 [10]. South Asians represent the largest visible minority group, and it is estimated that by 2031, 55% of Canada’s foreign-born population will report Asian origins [10,11]. Among these communities, Nepalese Canadians are one of the fastest-growing immigrant groups, with their population increasing by 54%, from 11,450 in 2011 to 21,380 in 2016, and continuing to rise [10,11]. Despite this growth, research on the Nepalese community in Canada remains limited. Existing evidence shows that Nepalese immigrants experience significant barriers to accessing healthcare and report unmet healthcare needs [12].

To the best of our knowledge, this is the novel study to examine delays in accessing and receiving primary care among Nepalese immigrants in Canada. This study explores factors associated with such delay among Nepalese immigrants residing in Calgary, Alberta.

## 2. Methods

### 2.1. Study Design and Setting

A community-based participatory research (CBPR) approach [13] was employed to conduct a cross-sectional study from January to June 2019, assessing delays in accessing healthcare and associated factors among Nepalese immigrants residing in Calgary, Alberta. The Nepalese Canadian community is one of the fastest-growing immigrant populations in Canada, with an estimated 7000 residents in Calgary, bringing distinct traditions, languages, and cultural identities. Grounded in the CBPR framework, Nepalese community scholars, citizen researchers, and local organizations were actively engaged throughout all study phases, including design, questionnaire development, recruitment, data interpretation, and knowledge translation. This collaboration enhanced cultural relevance, trust, and recruitment effectiveness while improving the credibility and applicability of study findings. The involvement of trusted community members facilitated identification of individual, social, and systemic barriers through a culturally informed lens.

### 2.2. Study Population and Data Collection

The study population comprised Nepalese immigrants 18-years or older living in Calgary who were able to read and write in English and willing to participate in the survey. Individuals with intellectual disability or any other condition that impeded communication or participation were excluded. We employed a non-probability convenience sampling approach as the primary recruitment method, complemented by snowball (referral) sampling leveraging existing social networks. These approaches were incorporated because no comprehensive sampling frame or centralized registry exists for Nepalese immigrants in Calgary. Recruitment was carried out in coordination with Nepalese Canadian Community Organizations (NCCOs) in Calgary, which supported the study by promoting it through social media channels, email networks, and word of mouth. NCCOs provided the research team with contact information for individuals, who expressed interest in participating in the study and completed the initial enrollment process. Participants who enrolled were encouraged to share study information within their personal and social networks, consistent with the snowball sampling method. Potential participants were informed of the study’s goals and objectives either in person or by phone by the research team. A self-administered questionnaire, along with an informed consent form, was distributed to eligible and interested participants either in person or via email. Completed questionnaires were returned directly to the research team. Follow-up reminders were made by telephone or in person two weeks after distribution for non-respondents. In total, 500 survey questionnaires were disseminated, of which 401 were completed and returned, yielding a response rate of 80.2%.

### 2.3. Sample Size Considerations

Due to the absence of a comprehensive sampling frame, probability-based sampling and conventional a priori power calculations were not feasible. Instead, sample size adequacy was assessed using Cochran’s formula as a benchmark for precision under conservative assumptions (95% confidence level, 5% margin of error, expected prevalence of 0.50). Applying finite population correction for an estimated population of approximately 7000 Nepalese residents in Calgary yielded a minimum required sample size of 365; the achieved sample of 401 participants exceeded this threshold.

### 2.4. Survey Tool

The survey questionnaire was developed based on a literature review and iterative input from the multidisciplinary research team and community scholars. The survey comprised 17 questions divided into two sections: (1) items related to sociodemographic characteristics and health status and (2) items addressing the outcomes of interest, such as delay seeking medical services, reasons for delay seeking care, and their potential impacts. Additional details about the survey items are described below. To enhance content validity, cultural relevance, clarity, and reliability, the survey was pilot tested with members of Nepalese community, leading to minor refinements in wording and structure.

### 2.5. Variable Measurements

Our outcome variable was a delay in seeking healthcare services. This referred to instances where an individual delayed (i.e., postponed) accessing healthcare, such as visiting a family physician, undergoing diagnostic tests, or receiving treatment necessary treatment. Delayed was measured using a self-reported single-item question: “During the past 12 months, was there any time that you had to delay seeking medical service within any of the following services?” The response to the question was recorded as binary “yes/no”. Participants who reported a delay were then asked to identify which PHC services were affected. A list of PHC items with corresponding binary (yes/no) response was compiled to determine the specific areas where delays occurred.

When participants reported experiencing a delay in seeking medical services, a follow-up question was asked to identify the reasons for the delay: “why did not they get care, or what were the reasons for the delay in seeking care?” The reported reasons were grouped into three categories: accessibility, availability, and acceptability, following [14]. In the context, “accessibility” refers to the ease of obtaining care, “availability” denotes the presence of sufficient and appropriate services, and “acceptability” signifies the cultural and social appropriateness of the services. Each category was represented as an indicator variable (presence or absence) derived by grouping related reasons for delay. The reasons for the delay in care were categorized into three groups. “Accessibility” included reasons such as cost or transportation/distance. “Availability” included reasons included such as ‘long wait time’ or ‘availability of services when requested’ or ‘services available in the area’. When the reasons for the delay in care were ‘too busy’ or ‘didn’t know where to get help’ or ‘felt it would be insufficient’ or ‘decided not to seek care’ or ‘negligence’ or ‘a language problem’ or ‘dislike the doctor/afraid,’ it was referred to as “acceptability”.

In addition, participants who reported delay in seeking care were asked a follow-up question to assess the impact of the delay in care: “did you feel any of the following impact(s) from that delay in your life?” Possible impacts were listed, with an open-ended “other” option included. Responses were recorded as binary (yes/no). Reported impacts were then grouped into two categories: personal impacts and economic impacts. Each category was represented as an indicator variable, reflecting the presence of absence of its respective impact, and was formed by combining related responses. The personal impact included outcomes such as “mental health”, “overall health impact”, “problem with daily activities”, suffering in personal relation”, “unable to provide childcare”, and “care unsatisfactory”. The economic impact indicator represents “increase cost of care”, “job lost”, “income lost”, “increased dependence”, “ordered medicine from back home”, and “increased use of over-the-counter drugs”.

We collected information on participants’ demographic, socioeconomic, and health-related characteristics to better understand factors associated with delays in seeking care. The survey included questions about age, sex, marital status, family size, education, employment status, and income. Age was categorized into six groups: under 25, 26–35, 36–45, 46–55, 56–65, and over 66 years. Sex was classified as male or female. Marital status was divided into three categories: married, single, or other (including separated, divorced, or widowed). Family size was grouped into three or fewer members and four or more members. Employment status was categorized as employed (full-time and part-time), unemployed, or student. Participants reported their annual household income, which was grouped into four categories: less than or equal to $25,000, $26,000–$50,000, $51,000–$75,000, and over $76,000. Participants were also asked whether they had a family doctor and extended health insurance coverage, as well as how long they had been in Canada (grouped into two categories: as ≤5 years or >5 years). Participants’ self-reported health rating was classified into three groups: excellent, very good and good, and fair and poor. Finally, participants were asked whether they had any chronic disease, with response categorized as yes or no. Sociodemographic and socioeconomic variables were categorized using commonly applied groupings in Canadian health research. Income categories reflected low-income thresholds reported in national literature and pragmatic considerations for analysis. Health status variables, including self-rated health and chronic disease presence, were self-reported using standard population health measures.

### 2.6. Statistical Analysis

Descriptive statistical analyses were conducted to summarize participants’ characteristics, with frequencies and percentages reported for categorical variables. Multivariable logistic regression was employed to examine statistical associations, rather than causal relationships, between delay in accessing care and sociodemographic and health-related factors. Variables included in the adjusted model were selected a priori based on established literature and theory on healthcare access among immigrant populations, reflecting known sociodemographic, economic, and health-related determinants that have been shown to influence access to care. Multicollinearity among predictors was assessed using the variance inflation factor (VIF), with all values <10 considered acceptable. Residuals and influence diagnostics, including deviance residuals and leverage, were also examined to identify potential outliers or influential observations. Adjusted odds ratios (AOR) with 95% confidence intervals (CIs) were computed while controlling sociodemographic characteristics and health status variables. Statistically significant was set at *p* < 0.05. A complete case analysis was conducted, excluding participants with missing”n outcome data, and sensitivity checks indicated no meaningful changes in key findings. Model fit was assessed comprehensively using multiple complementary approaches. Overall model significance was evaluated with the likelihood ratio test, comparing the full model to a null model with no predictors. Model calibration was evaluated using the Hosmer–Lemeshow goodness-of-fit test, which compares observed versus predicted event counts across deciles of predicted probabilities. A non-significant result did not show evidence of poor calibration. All analyses were performed using STATA version 14.2.

### 2.7. Ethics Approval

Ethics approval was obtained through the Conjoint Health Research Ethics Board at the University of Calgary (REB15-2325).

## 3. Results

### 3.1. Sociodemographic Characteristics of the Study Participants

Among the 401 survey respondents, there were almost an equal proportion of male (n = 202, 50.37%) and female (n = 199, 49.63%) participants. More than three-quarters of the respondents were between 26 and 45 years of age (n = 304, 75.81%) and married (n = 354, 88.28%). Nearly two-thirds (n = 252, 63%) reported having an education level equivalent to a bachelor’s degree or lower (including those who completed bachelor’s degree, college-level diploma or certificate, secondary school, or did not complete secondary school). Likewise, more than three-quarters of participants (n = 325, 81.05%) were employed, either full-time or part-time. Most respondents reported having a regular family doctor (n = 384, 95.76%) and extended health insurance coverage (n = 299, 74.56%). Over one-third (n = 133, 34.28%) indicated an annual household income between $51,000–$75,000. Furthermore, the majority of participants (n = 358, 91.79%) rated their health as excellent, very good, or good, while nearly one-third (n = 125, 31.17%) reported having at least one chronic disease. Details of participants’ sociodemographic characteristics are presented in Table 1.

### 3.2. Delayed Access to Care

Of the 401 survey participants, more than two-thirds (66.33%) reported experiencing a delay in accessing care during the 12 months preceding the study. The proportion of delays in care increased concurrently with advancing age, ranging from 59.38% in the ≤25 years age group to 92.31% in 56–65 years age group.

Regarding socioeconomic and demographic factors, delays were more prevalent among participants with an educational attainment of undergraduate level or below (69.84%) compared to those with a graduation degree (60.14%). Among employment categories, unemployed participants reported the highest proportion of delays (75.51%), while students reported the lowest (44.44%). Notably, high proportions of delays were reported by those with a family doctor (67.71%) as opposed to those without (35.29%). People with fair and poor health status reported a very high proportion of delays in accessing care (87.50%).

### 3.3. Factors Associated with More Likely to Have Delays in Accessing Care

Table 2 presents both the unadjusted and adjusted odds ratios describing the association between selected factors and delay in accessing care. A clear association was observed between age and delay in accessing care, with the likelihood increasing among older participants. Those aged 56–65 years were nearly eight times more likely to experience a delay in receiving care (UOR = 8.21; 95% CI: 0.95–71.09) compared to participants under 25 years of age. No significant differences were observed in delays based on marital status or family size. Participants with an education level of undergraduate or below had a 1.54 times higher likelihood of experiencing a delay (UOR = 1.54; 95% CI: 1.00–2.35) compared to those with graduate degrees. Interestingly, participants who had a family doctor were 3.84 times more likely to report delays in accessing care (UOR = 3.84; 95% CI: 1.39–10.63) than those without a family doctor. Participants residing in Canada for five years or longer had a modestly higher likelihood of experiencing a delay (UOR = 1.21; 95% CI: 0.79–1.85) compared to newer residents. Finally, those with one or more chronic conditions were nearly four times more likely to experience a delay in accessing care (UOR = 3.75; 95% CI: 2.20–6.40) compared to participants without chronic diseases.

After adjusting for covariates, delay in accessing care was strongly associated with participants’ age, with older individuals experiencing longer delays. Participants aged 56–65 years were nearly seven times more likely to report a delay in accessing care (AOR = 6.96; 95% CI: 0.42–115.39) compared to those under 25 years of age. Participants with an education level of undergraduate or below were 1.66 times more likely to experience a delay (AOR = 1.66; 95% CI: 0.95–2.93) than those with graduate degrees. Similarly, individuals who reported having a family doctor were over three times more likely to delay accessing care (AOR = 3.36; 95% CI: 1.02–11.00) than those without one. Participants with an annual household income of ≥$26,000 also had higher odds of delay (AOR = 1.47; 95% CI: 0.56–3.87) compared to those earning ≤$25,000. Moreover, participants who reported having one or more chronic diseases were more than four times more likely to experience a delay (AOR = 4.47; 95% CI: 2.25–8.89) compared to those without chronic conditions. Delays in accessing care were not statistically significant by sex, marital status, family size, employment status, extended health insurance coverage, or self-reported health status.

For the explanatory variables, multicollinearity was low, with VIF values ranging from 1.13 to 7.97 and a mean VIF of 2.62, indicating no evidence of problematic multicollinearity. The overall significance of the multivariable logistic regression model was confirmed by the likelihood ratio test (LR χ^2^(20) = 68.85, *p* < 0.001), indicating that the full model significantly improved fit compared with the null model. Model calibration was adequate, as shown by the Hosmer–Lemeshow test (χ^2^(8) = 6.26, *p* = 0.618).

### 3.4. Delayed in Accessing Care by a List of Healthcare Services

Table 3 presents the list of healthcare services in which participants experienced delays, along with their frequencies and percentages. Among the 266 participants who reported delays in care, the five most commonly affected areas were referrals and related services (69.17%), dental care (55.64%), family physician services (49.25%), vision care (40.60%), and laboratory services (37.22%). Other reported areas of delay included maternity and related services (9.40%), emergency hospital or urgent care services (6.02%), rehabilitation services (5.64%), child development services (3.01%), injury and treatment services (2.26%), and emergency medical or ambulance services, including 911 (0.75%).

### 3.5. Delayed Care by the Distribution of Reasons

More than two-thirds (66.33%) of participants reported delaying healthcare at least once in the 12 months preceding the survey, citing various reasons. The five most common reasons were long wait times (77.82%), cost or inability to afford care (55.64%), unavailability of care when needed (53.38%), lack of available services in the area (30.83%), and being too busy (30.19%). Other frequently cited reasons included transportation or distance barriers (29.70%), concerns that care would be inadequate (8.27%), language difficulties (7.89%), personal or family responsibilities (6.44%), not knowing where to seek help (4.89%), disliking or fearing the doctor (4.51%), deciding not to seek care (4.15%), and negligence (1.13%).

Further analysis revealed that “service availability” was the most common barrier, accounting for 84.59% (n = 225) of reported delays. “Service accessibility” issues, such as cost and transportation, were cited by 63.91% (n = 170) of participants, followed by “service acceptability,” which included personal beliefs and attitudes toward care, reported by 50.75% (n = 135). Table 4 presents detailed frequencies and percentages.

### 3.6. Self-Reported Impact Due to Care Delayed

Table 5 summarizes the self-reported impacts of care delays among Nepalese immigrants in Calgary. Participants who experienced delays described both personal and economic consequences due to their unmet healthcare needs. The most commonly reported personal impact was on mental health, including feelings of worry, anxiety, and stress, affecting not only participants themselves (74.72%) but also their family and friends (19.62%). Other frequently reported personal impacts included increased pain or symptoms (25.38%), worsening health problems (19.70%), overall health deterioration (10.19%), and dissatisfaction with received care (17.05%). Economic impacts were also common. The most frequently reported were increased use of over-the-counter medications (43.02%) and higher healthcare costs (20.38%). Additionally, some participants reported losing income (6.08%), losing work (4.17%), experiencing increased dependence on others (3.41%), or being unable to provide childcare (3.03%). Notably, 15.09% of participants reported importing or using medicines from their country of origin due to care delays.

## 4. Discussion

This study demonstrates a substantial burden of delays in accessing and receiving PHC services among Nepalese immigrants in Canada, with more than two-thirds of respondents reporting at least one delayed care in the preceding 12 months. The findings indicate that delays were associated with a combination of structural, socioeconomic, and health-related factors, rather than individual characteristics alone, particularly long wait times, unavailability of services when required, cost-related barriers, and competing work and family responsibilities. Importantly, delays were observed despite high levels of attachment to a family doctor, highlighting that formal attachment does not necessarily translate into timely access. Collectively, these findings point to gaps between universal health coverage and functional access to PHC for immigrant populations, with important implications for health equity.

The findings are broadly consistent with Canadian literature documenting barriers to timely care, including long wait times, service unavailability, and cost [5,15,16,17]. Similar patterns have been reported among Bangladeshi, Chinese, Punjabi, and other immigrant communities in Canada [18,19]. European studies also reported similar barriers among immigrants such as long wait times, language difficulties, and cost; however, many of them, particularly refugees, face additional challenges from deportation fears and digital exclusion that delay accessing care [20]. However, comparisons with national statistics and other studies should be interpreted cautiously, as differences in sampling strategies, populations, and outcome definitions may limit direct comparability. While the consistency of reported barriers across studies suggests shared structural challenges, methodological differences may influence the magnitude and distribution of reported delays. Although Canada’s publicly funded healthcare system is intended to ensure equitable access regardless of socioeconomic or immigration status [21], these findings underscore persistent gaps between policy intent and lived experience.

Several mechanisms may help explain the observed associations. The finding that individuals with a family doctor were more likely to report delays likely reflects health-system capacity constraints, such as limited appointment availability, high patient panel sizes, and referral bottlenecks, rather than improved access. Similar observations have been reported in studies examining unmet healthcare needs in Canada [14,22]. Older age and the presence of chronic disease were also associated with delayed access, potentially because individuals with greater healthcare needs require more frequent visits, referrals, and diagnostic services, increasing exposure to wait times. From a sociocultural perspective, older immigrants and those with chronic conditions may face additional barriers related to language, health system literacy, transportation, and reliance on family support [5,18,23]. These intersecting factors likely compound delays even when healthcare need is high. The impact of delayed care was evident across both personal and economic dimensions. Participants reported mental health challenges, including worry, anxiety, and stress, affecting not only themselves but also family members and friends. Physical consequences such as increased pain, worsening symptoms, and overall health deterioration were also described. These findings are consistent with prior studies documenting the adverse mental and physical health outcomes associated with delayed access care [21,22]. Financial consequences were similarly pronounced. Participants reported increased out-of-pocket costs, greater reliance on over-the-counter medications, and in some cases, importing medicines from their country of origin to manage health needs. These findings echo with earlier research showing that immigrants often seek medications and medical supplies outside Canada due to cost barriers [5]. Delays in accessing care were also associated with lost income, employment disruptions, and increased dependency, further compounding health and socioeconomic vulnerability. Comparable patterns have been reported among other immigrant and ethnic communities in Canada, including Bangladeshi, Chinese, and Punjabi populations [18,19].

A major strength of this study is its grounding in community-based participatory research (CBPR). Engagement with Nepalese community scholars, citizen researchers, and local organizations influenced survey design, recruitment, and interpretation of findings, fostering trust and enhancing cultural relevance. Community involvement enabled the identification of barriers and facilitators that may have been overlooked in conventional research [13,24], such as culturally specific perceptions of care, language challenges, and transportation preferences. By co-interpreting results, the research team ensured that analyses reflected lived experiences, improving validity and the applicability of findings to the community. However, important limitations must be acknowledged. The hybrid convenience and snowball sampling approach, relying on community organizations and social networks, may introduce selection bias. Initial recruits via open channels and organizations could over-represent more community-engaged or socially connected individuals, with participant referrals through social networks further amplifying homogeneity (e.g., similar demographics or integration levels). Consequently, the sample may under-represent isolated or less networked Nepalese immigrants, potentially reducing generalizability to the full population in Calgary. Inclusion was limited to English-literate participants, potentially excluding individuals with greater language barriers and underestimating access challenges. The cross-sectional design precludes causal or temporal inference, and reliance on self-reported data introduces recall and response bias. This study employed non-probability convenience sampling with snowball recruitment due to the absence of a comprehensive sampling frame for the population of interest. As a result, the findings may not be fully representative of all Nepalese residents in Calgary, and population-level generalizability is limited. In addition, while the achieved sample size provided reasonable precision for descriptive estimates within the study sample, formal a priori power calculations and inferential guarantees associated with probability-based sampling were not feasible. Associations observed in this study should therefore be interpreted as exploratory and considered in light of these limitations. Additionally, despite the overall sample size, some subgroup estimates were based on small numbers, resulting in wide confidence intervals and reduced precision. These results should be interpreted cautiously, as broad confidence intervals indicate greater uncertainty and limit the reliability of inference for some associations. Another potential limitation is that a screening log or denominator of individuals approached was not feasible with open recruitment channels (social media, posters, community networks), precluding formal assessment of non-response bias. This reflects the inherent challenges of broad, community-led outreach in immigrant communities where a sampling frame or population register is not available. Generalizability is therefore limited to Nepalese immigrants residing in Calgary and should not be extended to other immigrant groups or regions without caution.

## 5. Conclusions

In summary, delays in accessing care among Nepalese immigrants are shaped by multiple interrelated barriers, including long wait times, unavailability of services, financial constraints, transportation challenges, and competing personal responsibilities. These delays have tangible personal and economic consequences, contributing to health inequities and diminished well-being.

The findings indicate a need for targeted and locally actionable interventions. In the Calgary context, these include improving appointment availability and continuity within PHC practices serving immigrant-dense neighborhoods, strengthening referral coordination and follow-up processes, and embedding culturally and linguistically appropriate patient navigation supports within primary care settings. Given the prominence of wait times and system navigation challenges, policies addressing physician supply and service capacity [25] are particularly relevant.

Future research should include longitudinal and multi-city studies to assess changes in access over time and across regions, as well as qualitative follow-up studies to explore patient–provider interactions and decision-making processes underlying delays. Integrating survey findings with administrative health data could further clarify care pathways, validate self-reported delays, and identify points of system inefficiency. Such work is essential to moving beyond coverage toward equitable and timely access to primary healthcare for immigrant populations in Canada.

## Figures and Tables

**Table 1:** Characteristics of the study population (n = 401).

Variables	Delay in Accessing/Study Population	Proportion [95% CI]
**All cases**	266/401	66.33 [61.6–70.8]
**Sex**		
Male	133/202	65.84 [59.1–72.0]
Female	133/199	66.83 [60.0–73.0]
**Age**		
≤25 years	19/32	59.38 [42.3–74.5]
26–35 years	97/147	65.99 [58.0–73.2]
36–45 years	103/157	65.61 [57.9–72.6]
46–55 years	35/49	71.43 [57.6–82.2]
56–65 years	12/13	92.31 [66.7–98.6]
≥66 years	0/3	0.00 [0.0–56.1]
**Marital Status**		
Married	234/354	66.10 [61.0–70.8]
Single	29/42	69.05 [54.0–80.9]
Other (Separated/Divorced or Widowed)	3/5	60.00 [23.1–88.2]
**Family Size**		
3 & fewer members	145/204	71.08 [64.5–76.9]
4 & more members	121/195	62.05 [55.1–68.6]
**Educational Attainment**		
Graduation	89/148	60.14 [52.1–67.7]
Under graduation and below	176/252	69.84 [63.9–75.2]
**Employment status**		
Employed (FT + PT)	217/325	66.77 [61.5–71.7]
Unemployed	37/49	75.51 [61.9–85.4]
Student	12/27	44.44 [27.6–62.7]
**Family Doctor**		
Yes	260/384	67.71 [62.9–72.2]
No	6/17	35.29 [17.3–58.7]
**Extended Health Insurance Coverage**		
Yes	197/299	65.89 [60.3–71.0]
No	69/102	67.65 [58.1–75.9]
**Yearly household income**		
≤$25,000	27/46	58.70 [44.3–71.7]
≥$26,000–50,000	76/104	73.08 [63.8–80.7]
≥$51,000–75,000	86/133	64.66 [56.2–72.3]
≥$76,000	69/105	65.71 [56.2–74.1]
**Length of stay in Canada**		
Less than or equal to 5 years	111/174	63.79 [56.4–70.6]
5+ years	145/213	68.08 [61.5–74.0]
**Overall self-reported health rating**		
Excellent	15/36	41.67 [27.1–57.8]
Very good & good	212/322	65.84 [60.5–70.8]
Fair & poor	28/32	87.50 [71.9–95.0]
**Chronic diseases**		
Yes	105/125	84.00 [76.6–89.4]

Note: Total respondents for different variables vary due to missing data for respective variables.

**Table 2:** Unadjusted and adjusted odds ratio (OR) of delay in accessing care according to socio-demographic and health status.

Explanatory Variable	Unadjusted OR	95% CI	Adjusted OR	95% CI	Variance Inflation Factor (VIF)
**Sex**					
Male	Reference		Reference		
Female	1.05	[0.69–1.58]	0.86	[0.51–1.45]	1.23
**Age**					
≤25 years	Reference		Reference		
26–35 years	1.33	[0.61–2.91]	2.64	[0.70–9.96]	6.68
36–45 years	1.31	[0.60–2.84]	2.98	[0.70–12.63]	7.97
46–55 years	1.71	[0.67–4.37]	1.34	[0.26–6.91]	4.32
56–65 years	8.21	[0.95–71.09]	6.96	[0.42–115.39]	2.15
≥66 years	-	-	-	-	
**Marital Status**					
Married	0.87	[0.44–1.74]	0.39	[0.11–1.31]	2.34
Single	Reference		Reference		
Other (Separated/Divorced or Widowed)	0.67	[0.10–4.52]	0.11	[0.01–1.30]	1.41
**Family Size**					
3 & less members	Reference		Reference		
4 & more members	0.67	[0.44–1.01]	0.46	[0.26–0.81]	1.33
**Educational Attainment**					
Graduation	Reference		Reference		
Under graduation & below	1.54	[1.00–2.35]	1.66	[0.95–2.93]	1.33
**Employment status**					
Employed (FT + PT)	0.65	[0.33–1.30]	0.47	[0.17–1.27]	2.00
Unemployed	Reference		Reference		
Student	0.26	[0.10–0.71]	0.28	[0.08–1.01]	1.83
**Family Doctor**					
Yes	3.84	[1.39–10.63]	3.36	[1.02–11.00]	1.13
No	Reference		Reference		
**Extended health Insurance**					
Yes	0.92	[0.57–1.49]	0.95	[0.47–1.93]	1.57
No	Reference		Reference		
**Yearly household income**					
≤$25,000	Reference		Reference		
≥$26,000–50,000	1.91	[0.92–3.96]	1.47	[0.56–3.87]	3.01
≥$51,000–75,000	1.29	[0.65–2.56]	1.13	[0.42–3.06]	3.80
≥$76,000	1.35	[0.66–2.75]	1.66	[0.59–4.70]	3.63
**Length of stay in Canada**					
≤5 years	Reference		Reference		
5+ years	1.21	[0.79–1.85]	1.01	[0.58–1.77]	1.35
**Overall self-reported health rating**					
Excellent	0.10	[0.03–0.35]	0.13	[0.03–0.62]	2.65
Very good & good	0.28	[0.09–0.80]	0.36	[0.09–1.41]	2.71
Fair & poor	Reference		Reference		
**Chronic diseases**					
Yes	3.75	[2.20–6.40]	4.47	[2.25–8.89]	1.37

**Table 3:** The list of PHC elements that reported delay in accessing care (n = 266).

Number	List of Healthcare Services Delayed in Accessing	n (%)	95% CI
1	Accessing referral and related services	184 (69.17)	63.3–74.5
2	Dental and related services	148 (55.64)	49.5–61.6
3	Family physician (consultation/treatment)	131 (49.25)	43.0–55.5
4	Vision and related services	108 (40.60)	34.7–46.6
5	Laboratory related services	99 (37.22)	31.4–43.2
6	Rehabilitation and related services (therapy/social/medical/occupational)	15 (5.64)	3.3–9.0
7	Maternity care and related services (only female participants)	25 (9.40)	6.2–13.5
8	Basic emergency hospital/urgent care services	16 (6.02)	3.5–9.5
9	Medication/pharmacy and related services	15 (5.64)	3.3–9.0
10	Child developmental and related services (immunization/growth of child)	8 (3.01)	1.3–5.9
11	Injury treatment-related service	6 (2.26)	0.8–4.9
12	Emergency Medical Ambulance Service, 911 service	2 (0.75)	0.1–2.7

Note: Respondents could report more than one impact resulting from delays in accessing care; therefore, the percentages in this table are not additive and may sum to more than 100%. 95% confidence intervals (CIs) for proportions were calculated using the normal approximation for counts greater than 10 and the exact (Clopper–Pearson) method for small counts (≤10).

**Table 4:** Reasons for the delay in accessing care (n = 266).

Variables	n (%)	95% CI
**Accessibility**	170 (63.91)	58.0–69.8
Cost/Cost I could not afford to pay	148 (55.64)	49.5–61.6
Distance/transportation	79 (29.70)	24.2–35.1
**Availability**	225 (84.59)	80.3–89.7
Waiting time too long	207 (77.82)	72.9–82.7
Care not available when requested	142 (53.38)	47.3–59.6
Care is not available in the area	82 (30.83)	24.5–36.1
**Acceptability**	135 (50.75)	44.8–56.7
Too busy	80 (30.19)	24.6–35.5
Felt it would be inadequate	22 (8.27)	5.3–11.9
Language problem	21 (7.89)	5.1–11.6
Personal/family responsibility	17 (6.44)	3.8–10.1
Didn’t know where to go/get help	13 (4.89)	2.7–8.3
Dislike doctor/afraid	12 (4.51)	2.5–7.9
Decided not to seek care	11 (4.15)	2.2–7.4
Did not get around it/did not bother/negligence	3 (1.13)	0.2–3.2
Other reason(s), please specify……	3 (1.13)	0.2–3.2
Accessibility + Availability	256 (96.24)	94.0–97.9
Accessibility + Acceptability	218 (81.95)	77.2–86.2
Availability + Acceptability	237 (89.10)	85.3–93.0

Note: Respondents could report more than one impact resulting from delays in accessing care; therefore, the percentages in this table are not additive and may sum to more than 100%. 95% confidence intervals (CIs) for proportions were calculated using the normal approximation for counts greater than 10 and the exact (Clopper–Pearson) method for small counts (≤10).

**Table 5:** Impact of delay in accessing care (n = 266).

Variables	n (%)	95% CI
**Personal Impacts**		
Worry, anxiety, stress	198 (74.72)	69.1–79.7
Pain or other symptoms	67 (25.38)	20.2–30.6
Worry or stress for family or friends	52 (19.62)	14.7–24.6
Health problem increased	52 (19.70)	14.7–24.7
Care unsatisfactory?	45 (17.05)	12.5–21.6
Overall health deteriorated condition/got worse	27 (10.19)	6.7–14.3
Problems with activities of daily living	10 (3.80)	1.8–6.9
Personal relationships suffered	7 (2.64)	1.1–5.3
Other impact(s), please specify…….	1 (0.38)	0.01–2.1
**Economic Impacts**		
Increased use of over-the-counter drugs	114 (43.02)	36.7–49.5
Use medicine that is brought from back home	40 (15.09)	10.8–20.0
Loss of income	16 (6.08)	3.4–9.6
Loss of work	11 (4.17)	2.1–7.4
Increased dependence	9 (3.41)	1.6–6.3
Unable to do (much) childcare	8 (3.03)	1.3–5.

Note: Respondents could report more than one impact resulting from delays in accessing care; therefore, the percentages in this table are not additive and may sum to more than 100%. 95% confidence intervals (CIs) for proportions were calculated using the normal approximation for counts greater than 10 and the exact (Clopper–Pearson) method for small counts (≤10).

## Data Availability

The data presented in this study are available on request from the corresponding author due to ethical restrictions. The Research Ethics Board approval under which the data were collected (REB15-2325, 11 January 2016) does not permit sharing individual-level data, as participants were not informed of data sharing at the time of consent. Therefore, only aggregated data may be shared.
